# Supplementary material for: Elimination of fukutin reveals cellular and molecular pathomechanisms in muscular dystrophy-associated heart failure
Source: Nat Commun. 2019 Dec 17;10:5754. doi: 10.1038/s41467-019-13623-2 (PMC6917736; doi:10.1038/s41467-019-13623-2)
Supplement: Supplementary file 1 — Supplementary Infomation [file 41467_2019_13623_MOESM1_ESM.pdf]

Elimination of fukutin reveals cellular and molecular pathomechanisms in muscular dystrophy-associated heart failure

Yoshihiro Ujihara[1,2,4], Motoi Kanagawa[3], Satoshi Mohri[1,2], Satomi Takatsu[1], Kazuhiro Kobayashi[3], Tatsushi Toda[3], Keiji Naruse[1], Yuki Katanosaka[1*]

[1]Department of Cardiovascular Physiology, Graduate School of Medicine, Dentistry and Pharmaceutical Sciences, Okayama University, Shikata-cho 2-7-1, Okayama City, Okayama 7008558, Japan
[2]Department of Physiology, Kawasaki Medical School, Matsushima 577, Kurashiki City, Okayama 7010192, Japan
[3]Division of Neurology/Molecular Brain Science, Kobe University Graduate School of Medicine, Kobe 650-0017, Japan
[4]Department of Electrical and Mechanical Engineering, Graduate School of Engineering, Nagoya Institute of Technology, Nagoya 466-8555, Japan (Present affiliation of Y.U.)

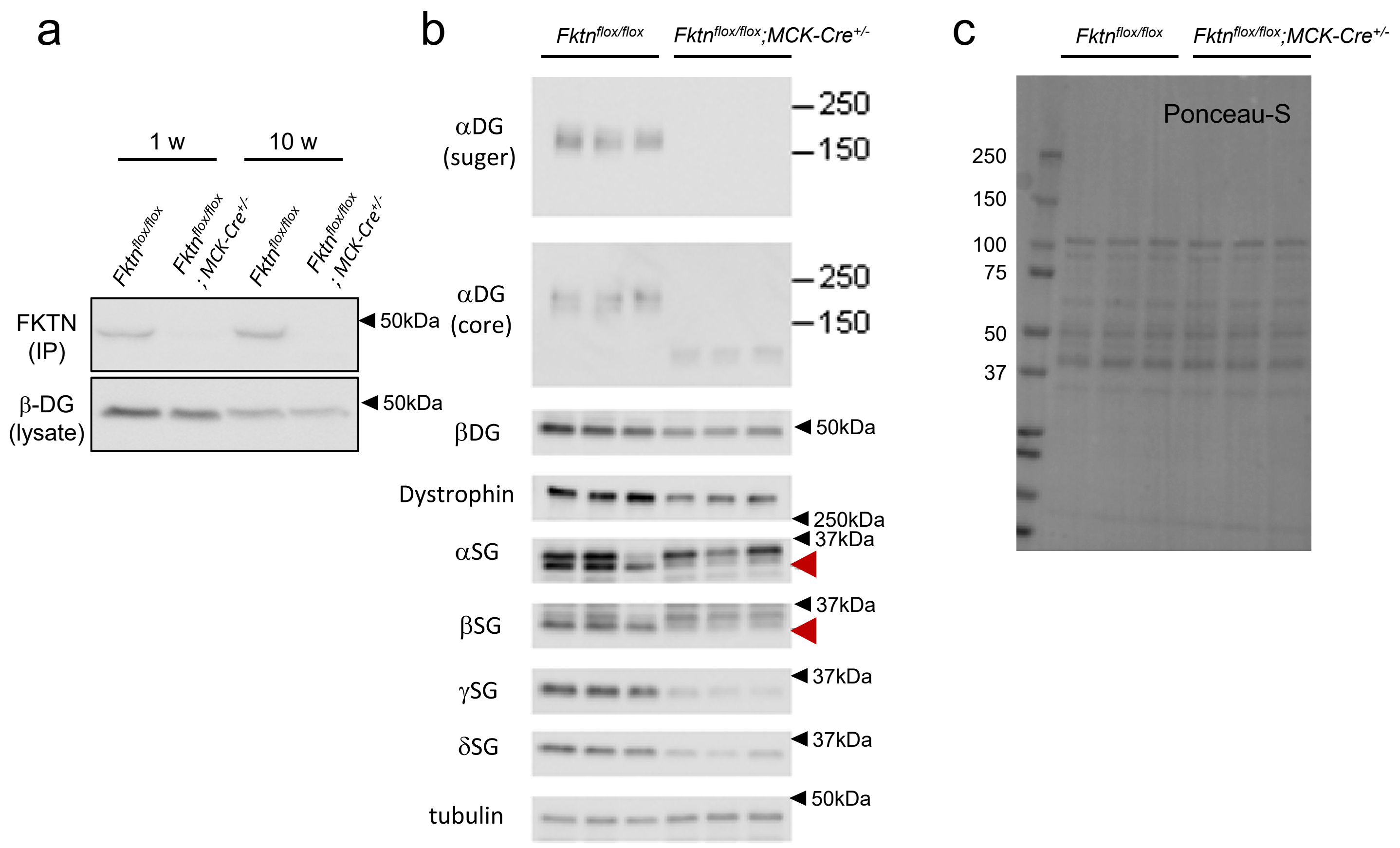

Supplementary Figure 1. Reduction of α-DG glycosylation and expression of DGC complex in floxed control (*Fktn*$^{flox/flox}$) and MCK-*Fktn*-cKO (*Fktn*$^{flox/flox}$*;MCK-Cre*$^{+/-}$) hearts.

(**a**) Representative immunoblot showing the lack in FKTN expression by anti-FKTN-immunoprecipitation in samples from MCK-*Fktn*-cKO hearts. β-DG was used as the loading control. (**b**) Representative immunoblot showing the effects of *Fktn* elimination in DGC proteins of MCK-*Fktn*-cKO hearts. N=3 hearts per group. (c) For loading controls, a portion (15 μg) of the solubilized samples was stained with Ponceau-S (right panel).

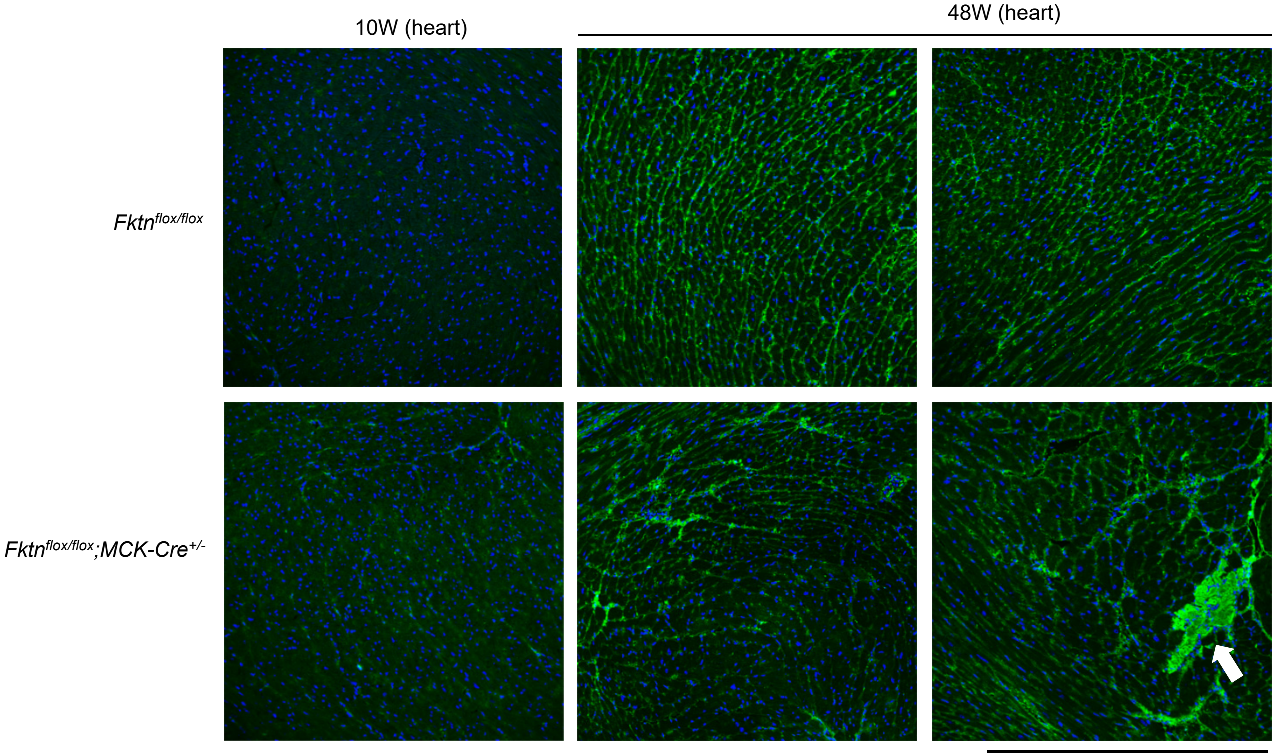

Supplementary Figure 2. the detection of cell damage in floxed control (*Fktn*$^{flox/flox}$) and MCK-*Fktn*-cKO (*Fktn*$^{flox/flox}$*;MCK-Cre*$^{+/-}$) hearts of 10- and 48-weeks-old mice.

Representative images showing endogenous IgG (green) in floxed control (*Fktn*$^{flox/flox}$) and MCK-*Fktn*-cKO (*Fktn*$^{flox/flox}$*;MCK-Cre*$^{+/-}$) hearts of 10 and 48 weeks old mice. MCK-*Fktn*-cKO (*Fktn*$^{flox/flox}$*;MCK-Cre*$^{+/-}$) hearts show the patches of IgG-positive myocytes consisted of contacting neighboring cells. Scale bar, 500 μm.

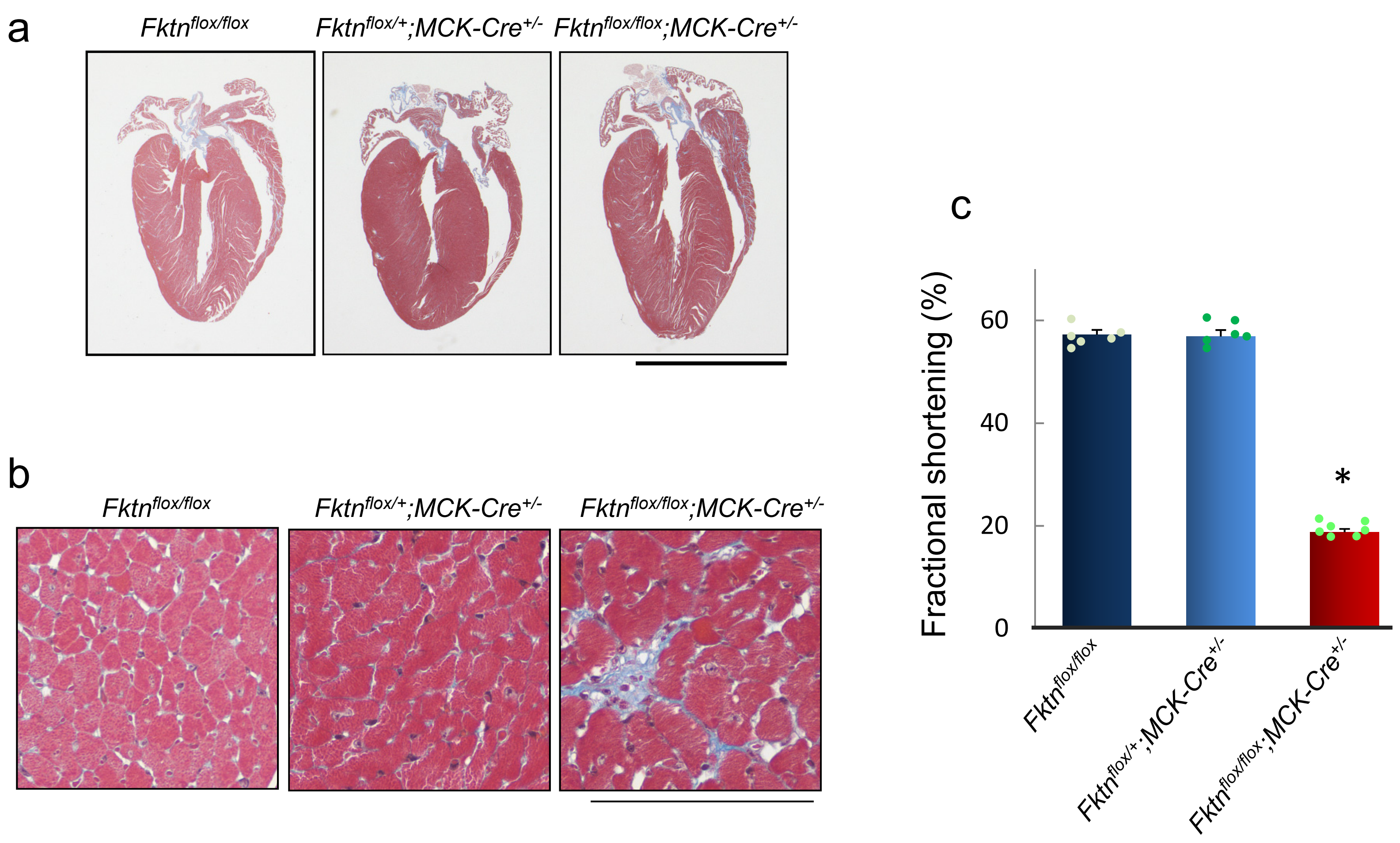

Supplementary Fig. 3: Change in cardiac morphology, histology and pump function in 48weeks-old *Fktn*$^{flox/flox}$ (Floxed control), *Fktn*$^{flox/+}$;*MCK-Cre*$^{+/-}$(hetero) and *Fktn*$^{flox/flox}$;*MCK-Cre*$^{+/-}$ (MCK-*Fktn*-cKO) mice.

(**a**) Cardiac morphology. Scale bar, 5 mm. (**b**) Masson's trichrome staining of left ventricle. Scale bar, 100 μm. (**c**) Change in cardiac function (n=6 per group). Data are expressed as means ± s.e.m. Asterisk, P<0.05 vs. *Fktn*$^{flox/flox}$ (Floxed control) based on Dunnett's *post hoc* tests.

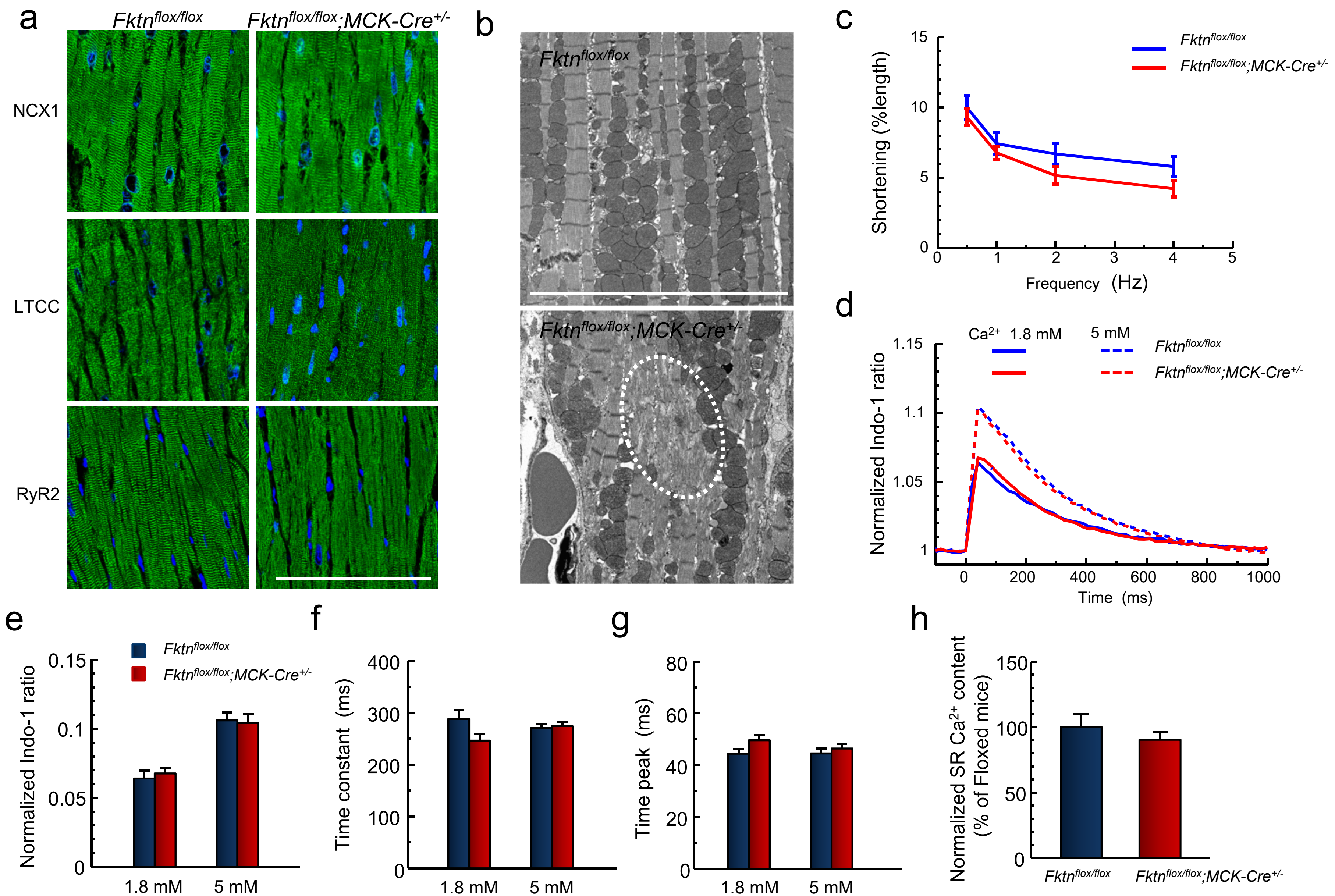

Supplementary Figure 4. Normal cardiomyocyte functioning in young adult MCK-*Fktn*-cKO (*Fktn*$^{flox/flox}$*;MCK-Cre*$^{+/-}$) mice. (**a**) Representative immunostaining for NCX1, RyR2, and LTCC (DAPI counterstain, blue) in hearts of 10-week-old mice. Scale bar, 100 μm (**b**) Representative electron micrographs of myofilaments. Dashed circle indicates the disordered area. Scale bar, 10 μm. (**c**) Frequency-dependent shortening of cardiomyocytes (*n* = 26 and 28 cells from 4 hearts per group). (**d**) Indo-1 fluorescence in single cardiomyocytes stimulated at 1 Hz (1.8 mM Ca^{2+}, *n* = 17 and 30 cells from 3 and 4 hearts, respectively ; 5 mM Ca^{2+}, *n* = 25 and 35 cells from 3 hearts per group). Peak amplitudes (**e**) decay time constants (obtained by fitting the decline phase) (**f**) and times to peak (**g**) of Ca^{2+} transients. (**h**) Estimation of sarcoplasmic reticulum (SR) Ca^{2+} content (*n* = 11 and 21 cells from 2 and 3 hearts, respectively). Data are means ± s.e.m.

Supplementary Figure 4 Ujihara et al.,

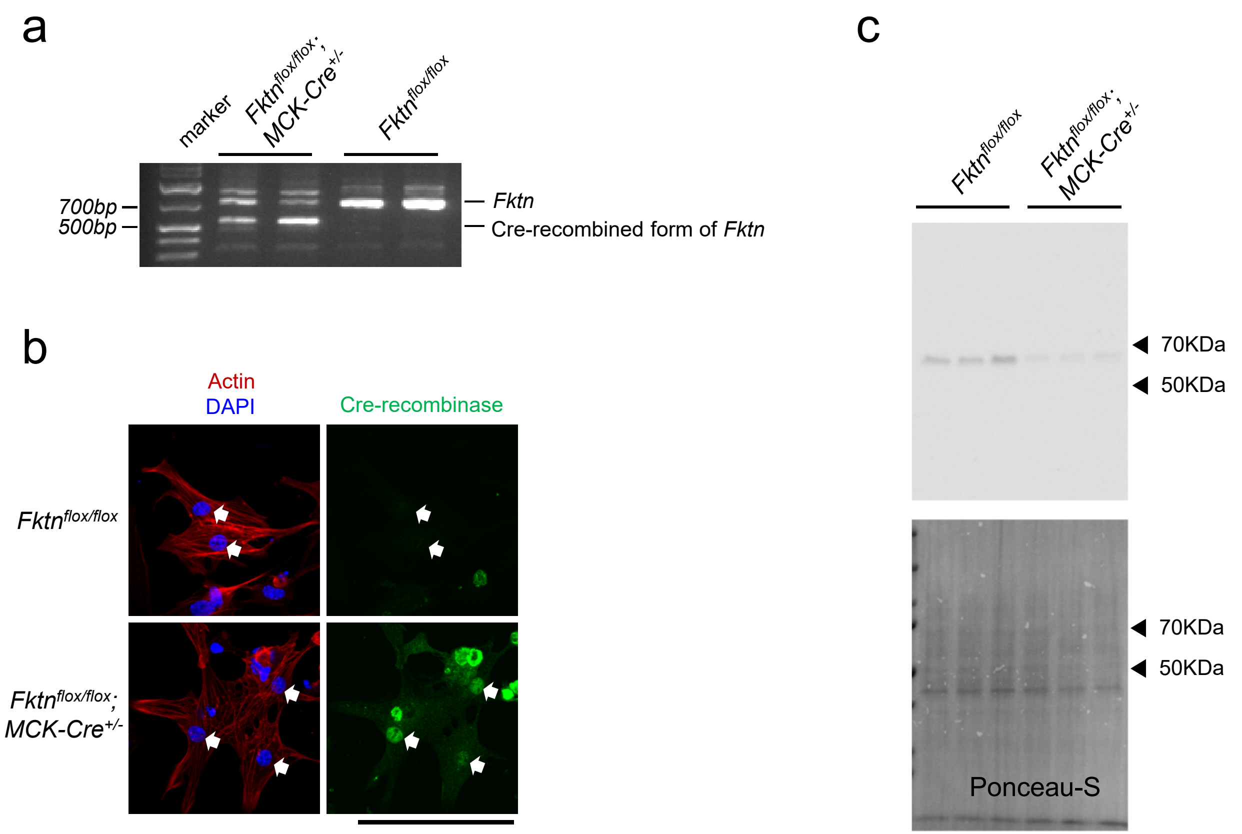

Supplementary Fig. 5: Cre-recombination, the exppresion of Cre-recombinase in isolated cardiomyocytes of newborn MCK-*Fktn*-cKO (*Fktn*$^{flox/flox}$;*MCK-Cre*$^{+/-}$) **mice.** (**a**) PCR fragments of *Fktn* mRNA. Cre–recombined form of Fktn was observed in myocytes isolated from newborn MCK-*Fktn*-cKO (*Fktn*$^{flox/flox}$;*MCK-Cre*$^{+/-}$) mice cultured for 48 hour. Fktn exon 2, which contains the initial ATG, was flanked by 2 loxP sequences (Kanagawa et. al., Hum Mol Genet 2013), and thus Cre-recombination produces non-functional transcript. (**b**) Representative fluorescent image (Red; Actin, Green; Cre-reombinase, Blue; DAPI) of cultured neonatal cardiomyocytes isolated from Floxed-*Fktn* and MCK-*Fktn*-cKO (*Fktn*$^{flox/flox}$;*MCK-Cre*$^{+/-}$) mice (at 48 hour after isolation). White arrow showed the nuclear. The neonatal cultured myocytes of MCK-*Fktn*-cKO (*Fktn*$^{flox/flox}$;*MCK-Cre*$^{+/-}$) showed the expression of Cre-recombinase in nuclear. Scale bar, 100 μm. (**c**) Representative immunoblot showing the reduction in FKTN expression by anti-FKTN-immunoprecipitation in samples from 1-day old MCK-*Fktn*-cKO (*Fktn*$^{flox/flox}$;*MCK-Cre*$^{+/-}$) mice (upper panel). For loading controls, a portion (10 μg) of the solubilized samples that were subjected to FKTN enrichment was stained with Ponceau-S (left, lower panel).

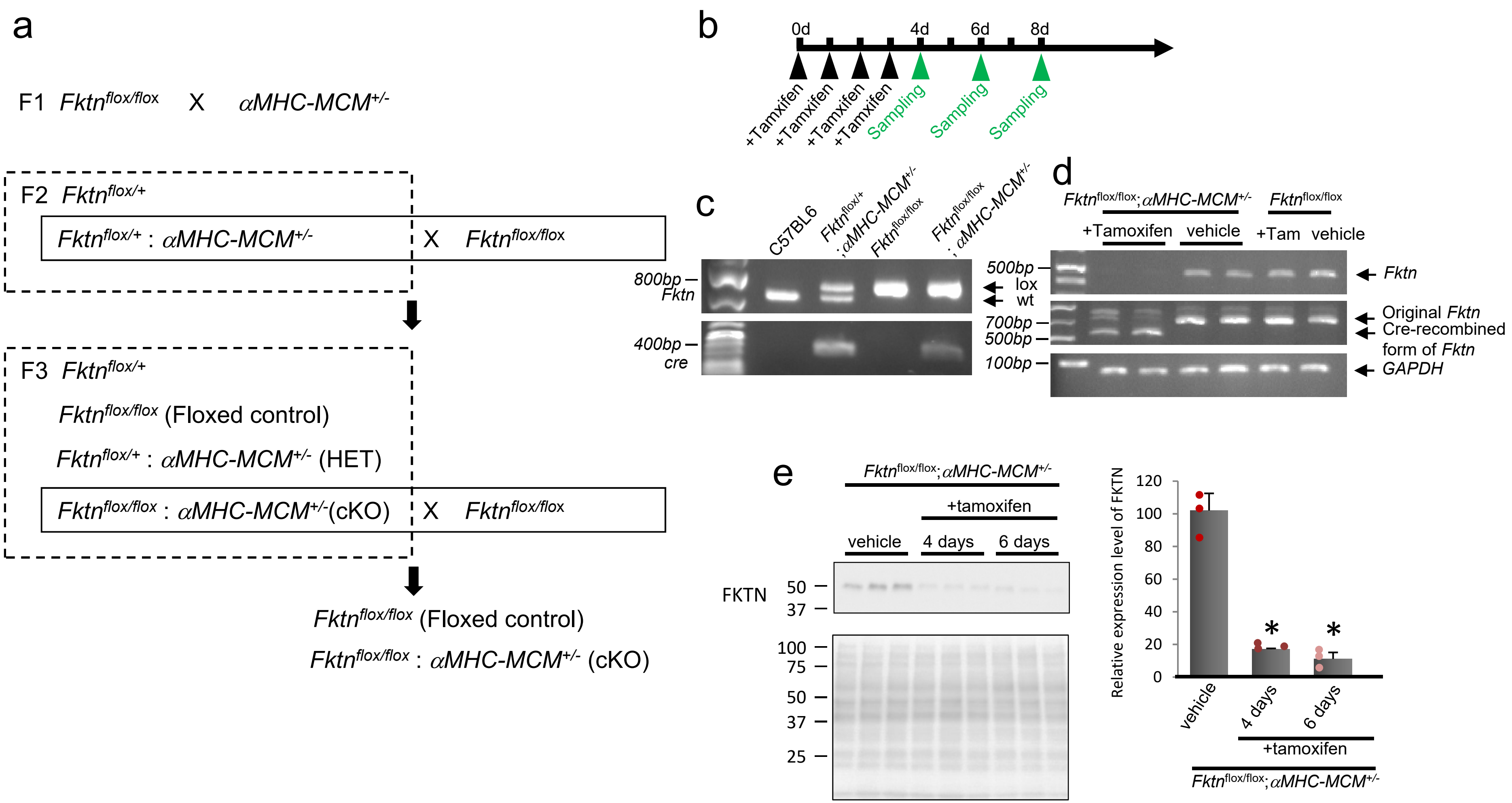

Supplementary Fig. 6: Generation of αMHC-MCM-*Fktn*-cKO (*Fktn*$^{flox/flox}$;*αMHC-MCM*$^{+/-}$) mice.
(**a**) Crossbreeding strategy for generation of the conditional *Fktn*-knockout mice (cKO). Littermates of generation F3 were used as controls and cKO in all the experiments. (**b**) Schedule of tamoxifen-treatment and sample collection. To induce Cre-mediated recombination, we injected *Fktn*$^{flox/flox}$;*αMHC-MCM*$^{+/-}$, *Fktn*$^{flox/+}$;*αMHC-MCM*$^{+/-}$, *Fktn*$^{flox/flox}$ mice intraperitoneally with 8 mg/kg tamoxifen or vehicle once daily for four consecutive days. (**c**) Genotyping PCR of mouse genomic DNA. (top) PCR fragments including the loxP insertion site on *Fktn* locus: 778-bp fragment indicates the wild-type *Fktn* and the 870-bp fragment indicates the insertion of loxP. (bottom) PCR fragments of the Cre recombinase gene. (**d**) Representative PCR image showing the PCR fragments of Fktn in *Fktn*$^{flox/flox}$;*αMHC-MCM*$^{+/-}$ and *Fktn*$^{flox/flox}$ hearts with or without 4 days of tamoxifen-treatment. Expression level of *Fktn* mRNA was confirmed by PCR using the primer pair Ex1F1, 5′- TACCCTGAGTAGCGGTCGTC -3′ and Ex2R2, 5′- GTGCTTGTAGTAGTACAGCT -3′, yielding products of 460 bp (upper panel). Cre-recombibined form of *Fktn* was confirmed by PCR using Ex1F1 and Ex4R1, 5′- ACAGGTGATACTGCAGTGCG -3′, yielding products of 523 bp (Cre-recombined form of *Fktn*) and 726 bp (original *Fktn*). (**e**) Representative immunoblot showing the reduction in FKTN expression by anti-FKTN-immunoprecipitation in samples from αMHC-MCM-*Fktn*-cKO hearts treated for 4 days with tamoxifen (left, upper panel). We treated αMHC-MCM-*Fktn*-cKO mice with tamoxifen or vehicle for 4 consecutive days, then carried out analysis at different time point. For loading controls, a portion (40 μg) of the solubilized samples that were subjected to FKTN enrichment was stained with Ponceau-S (left, lower panel). Relative expression level of FKTN expression (right panel, n=3 mice per group). Asterisk, $p< 0.05$ vs. vehicle treated αMHC-MCM-*Fktn*-cKO mice on Dunnett's *post hoc* tests.

Supplementary Figure 6 Ujihara et al.,

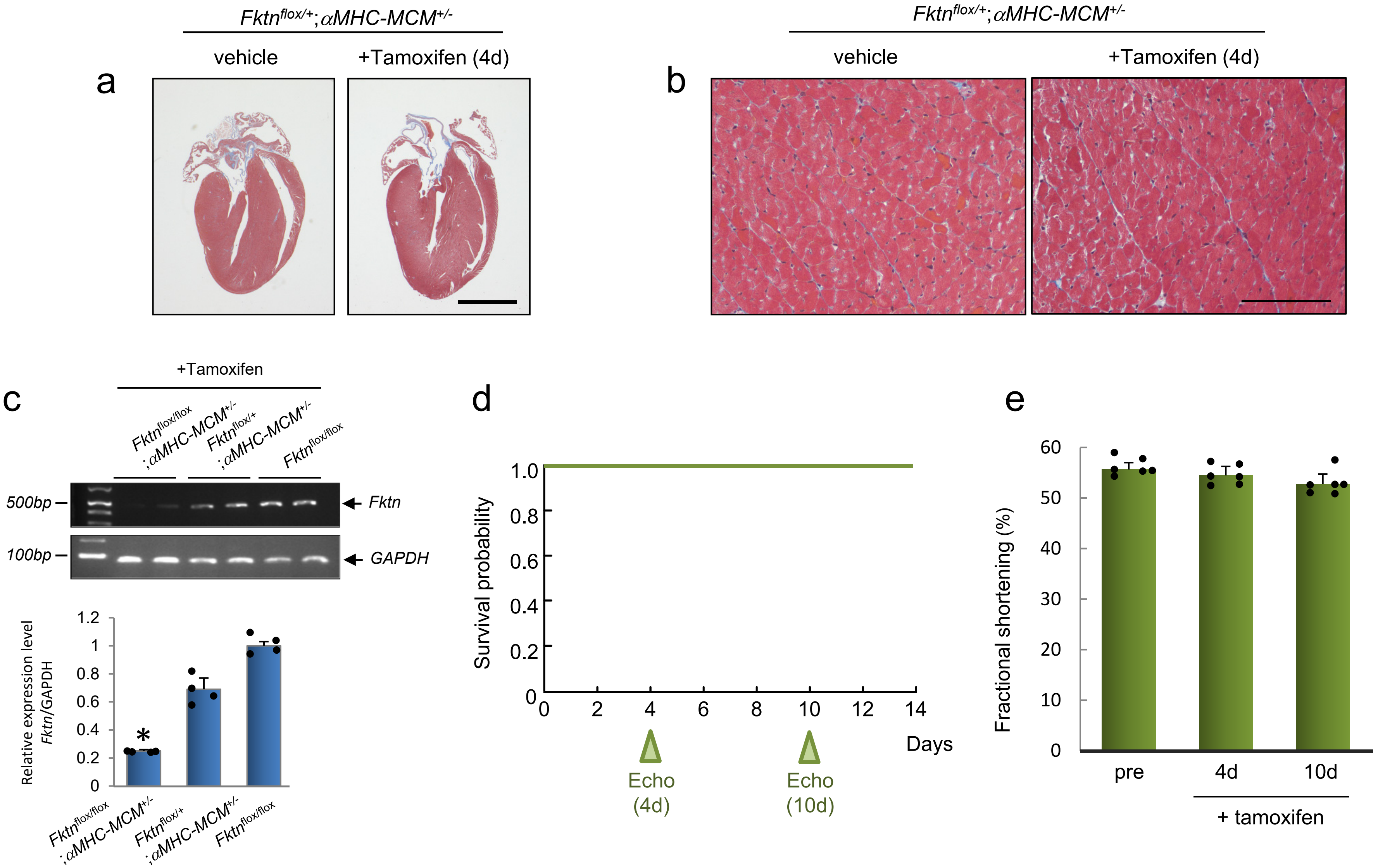

Supplementary Fig. 7: No change in cardiac morphology, histology and pump function after tamoxifen treatment (i.p. injection for four consecutive days at 8 mg/kg body weight) in C57BL/6-congenic *Fktn*$^{flox/+}$;*αMHC-MCM*$^{+/-}$ (hetero) mice.
(a) Cardiac morphology. Scale bar, 5 mm. (b) Masson's trichrome staining of left ventricle. Scale bar, 100 μm. (c) Relative expression of Fktn mRNA in the heart of tamoxifen-treated *Fktn*$^{flox/flox}$;*αMHC-MCM*$^{+/-}$, *Fktn*$^{flox/+}$;*αMHC-MCM*$^{+/-}$, *Fktn*$^{flox/flox}$ mice (N=4 hearts from each group). Expression level of *Fktn* mRNA was confirmed by PCR using the primer pair 5′- TACCCTGAGTAGCGGTCGTC-3′ and 5′- GTGCTTGTAGTAGTACAGCT-3′, yielding products of 460 bp. *P<0.05 vs. *Fktn*$^{flox/flox}$ (Floxed control) based on Dunnett's *post hoc* tests. (d) Survival properties (N=14). (e) No change in cardiac pump function for pre-treatment (pre) versus tamoxifen-treated *Fktn*$^{flox/+}$; αMHC-MCM$^{+/-}$ mice, for 10 days after onset of treatment (n=6), Data are expressed as means ± s.e.m.

Supplementary Figure 7 Ujihara et al.,

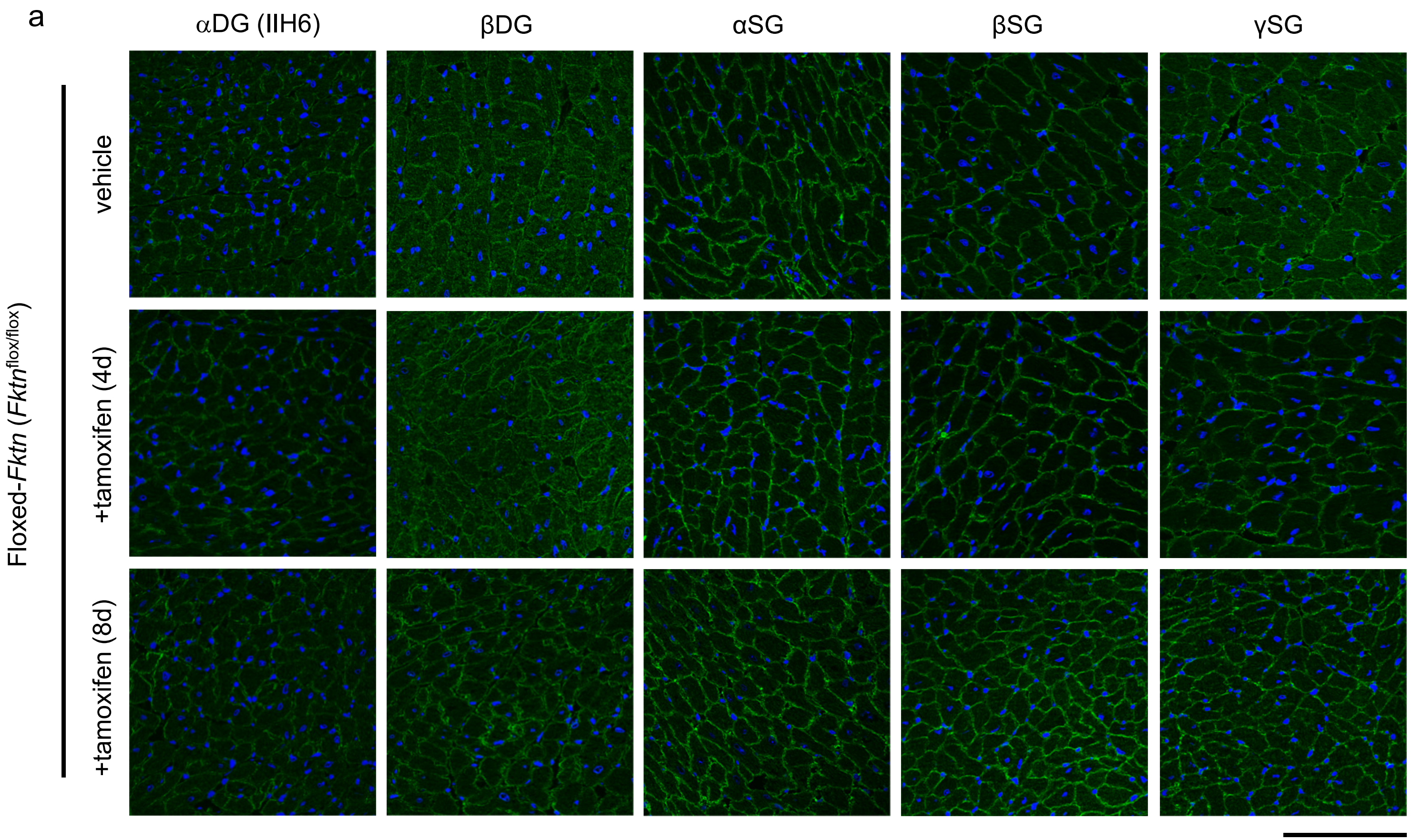

Supplementary Fig. 8: Changes in expression and localization of αDG (IIH6), βDG, αSG, βSG and γSG in Floxed-*Fktn* (*Fktn*$^{flox/flox}$), αMHC-MCM-*Fktn*-cKO (*Fktn*$^{flox/flox}$;*αMHC-MCM*$^{+/-}$) and *Fktn*$^{flox/+}$;*αMHC-MCM*$^{+/-}$ (hetero) hearts with or without tamoxifen.
We treated Floxed-*Fktn* (*Fktn*$^{flox/flox}$), αMHC-MCM-*Fktn*-cKO (*Fktn*$^{flox/flox}$;*αMHC-MCM*$^{+/-}$) and *Fktn*$^{flox/+}$;*αMHC-MCM*$^{+/-}$ (hetero) mice with tamoxifen or vehicle for 4 consecutive days, then carried out analysis at different time point. Representative immunofluorescent images of DGC proteins. (a) In the hearts of treated Floxed-*Fktn* (*Fktn*$^{flox/flox}$) mice with or without tamoxifen, the glycosylated form of DG and other DGC proteins show membrane localization at the sarcolemma. Scale bar, 100 μm.

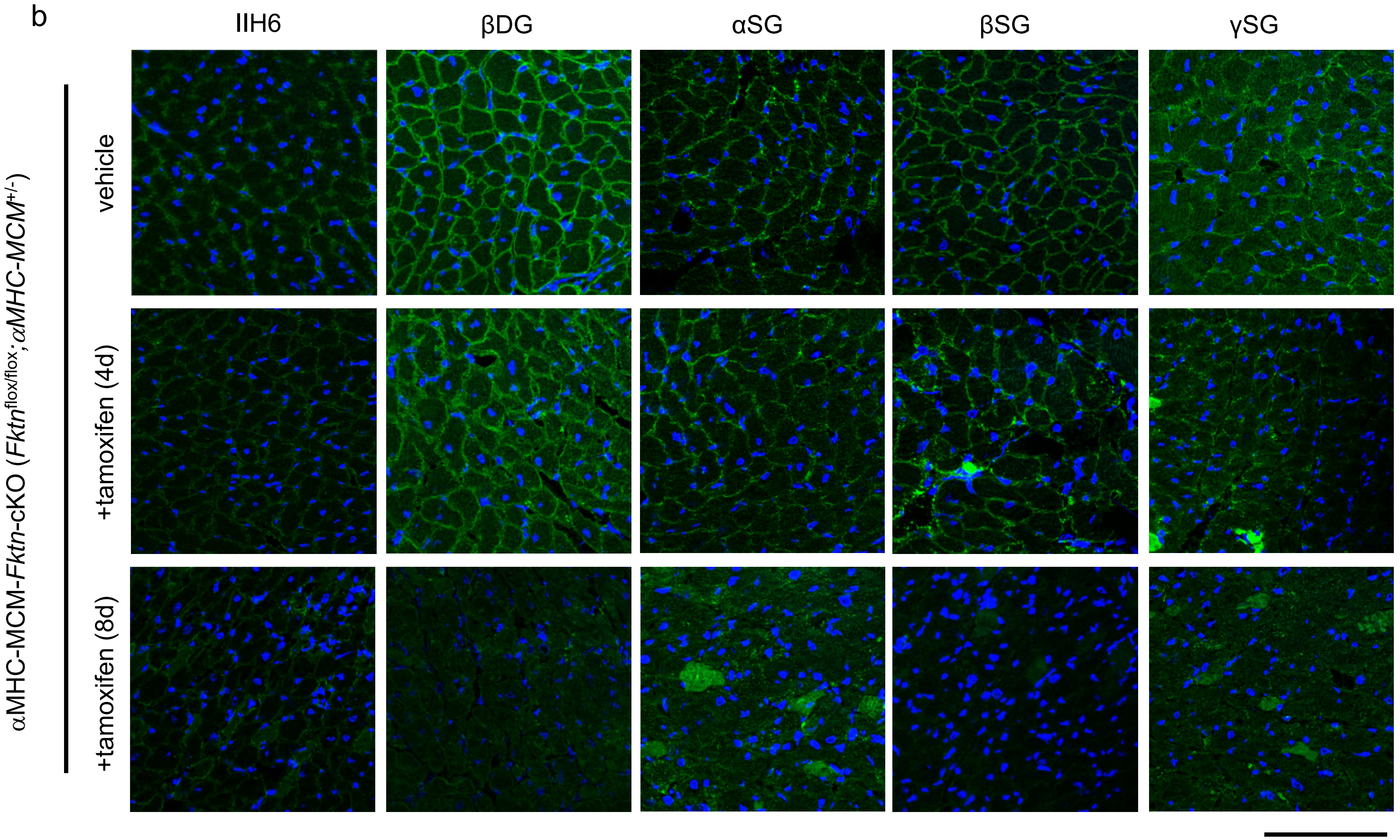

Supplementary Fig. 8 (b): In the hearts of αMHC-MCM-*Fktn*-cKO (*Fktn*$^{flox/flox}$;*αMHC-MCM*$^{+/-}$) mice treated for 4 days with tamoxifen, the glycosylated form of DG and other DGC proteins show membrane localization at the sarcolemma. Eight days after the onset of tamoxifen treatments (8d), the membrane localization of these proteins disappear. Scale bar, 100 μm.

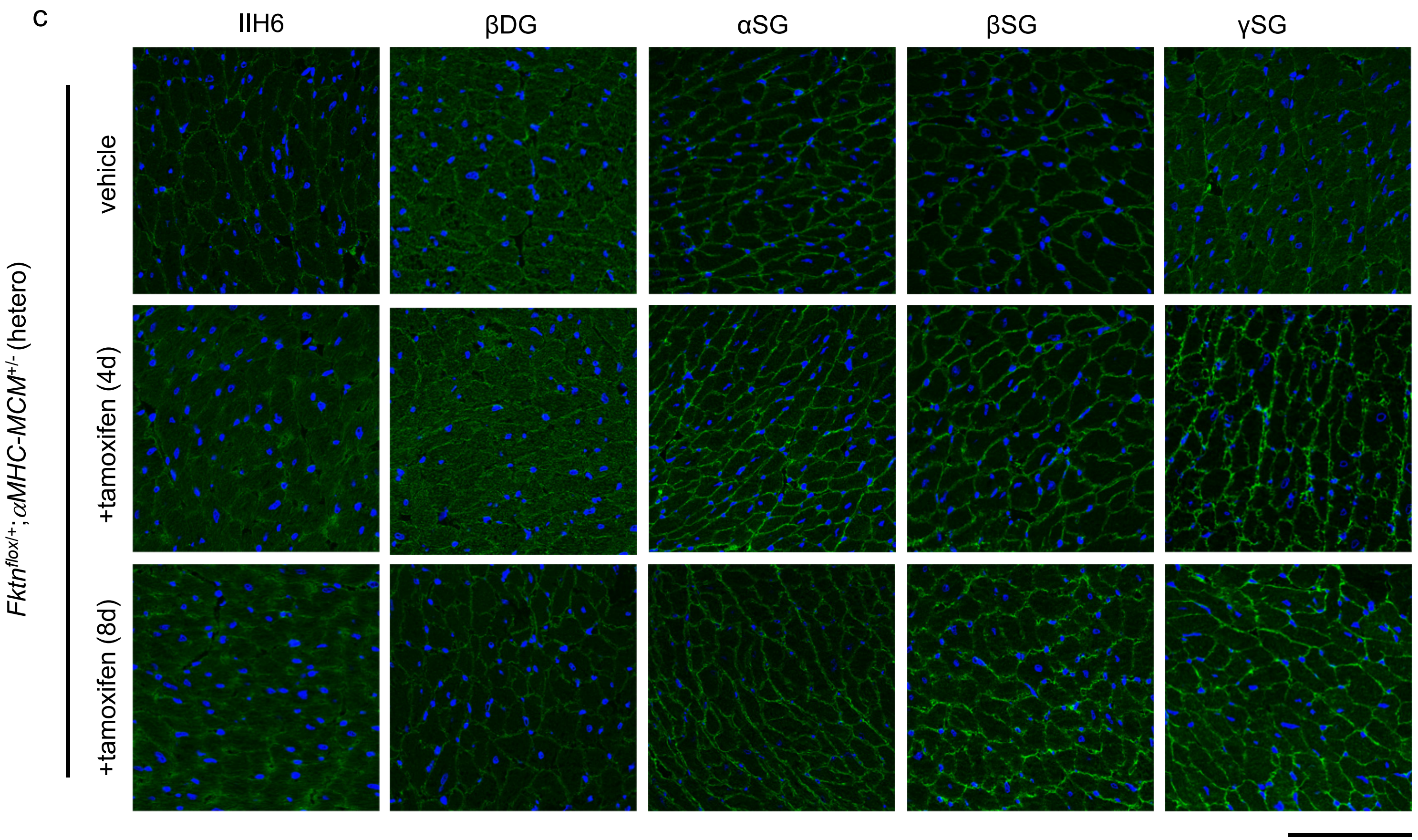

Supplementary Fig. 8 (c) : In the hearts of treated *Fktn*$^{flox/+}$;α*MHC-MCM*$^{+/-}$ (hetero) mice with or without tamoxifen, the glycosylated form of DG and other DGC proteins show membrane localization at the sarcolemma. Scale bar, 100 μm.

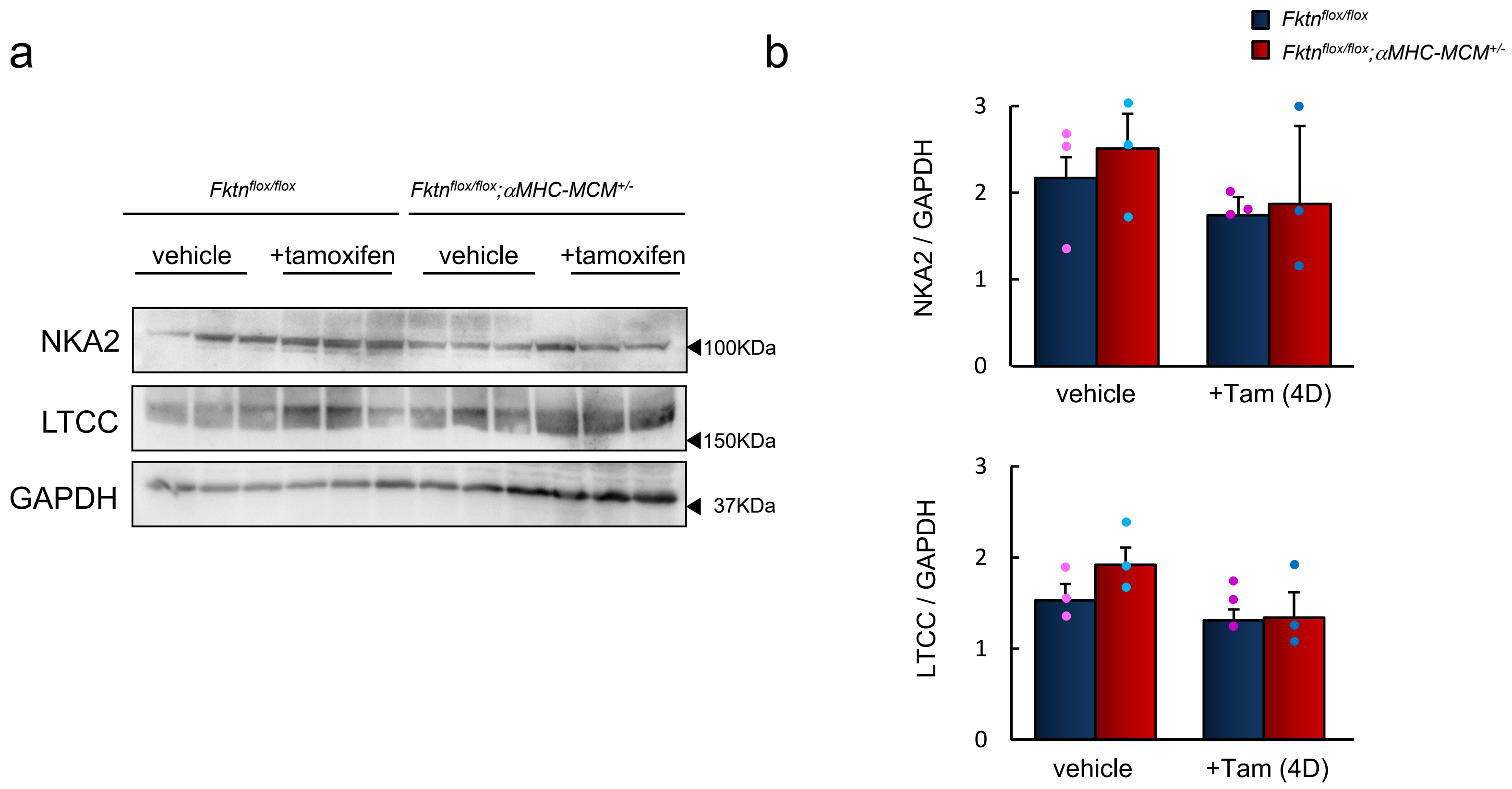

Supplementary Fig. 9: Changes in NKA2 and LTCC expression in floxed (*Fktn*$^{flox/flox}$) and αMHC-MCM-*Fktn*-cKO (*Fktn*$^{flox/flox}$;*αMHC-MCM*$^{+/-}$) hearts with or without tamoxifen.
(**a**) Representative immunoblot showing expressions of Na^+/K^+ ATPase 2 (NKA2) and LTCC normalized to GAPDH in floxed and cKO hearts (N=3 per group); 10 mg protein extracts were loaded in each lane. (**b**) Expression levels of each protein.

Supplementary Figure 9 Ujihara et al.,

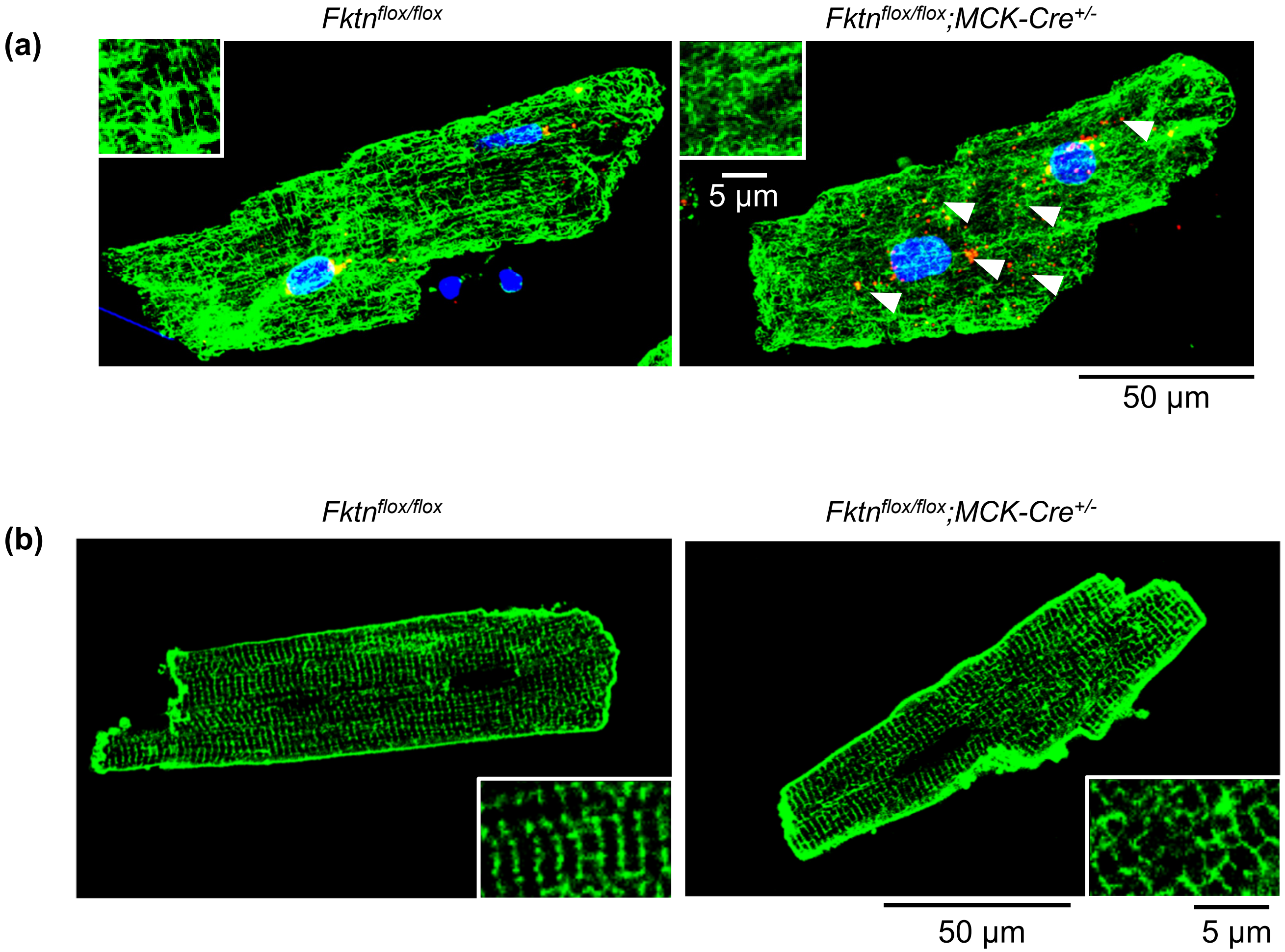

Supplementary Fig. 10: Changes in localization of GM130 and accumulation of microtubule in floxed (*Fktn*$^{flox/flox}$) and MCK-*Fktn*-cKO (*Fktn*$^{flox/flox}$;*MCK-Cre*$^{+/-}$) hearts at 10 month after birth.

(**a**) Representative immunofluorescence images for MT (green), GM130 (red) and DAPI (blue) staining. Arrow heads show abnormally distributed GM130 signals in MCK-cKO myocytes. Scale bar, 50 µm. (**b**) Representative membrane staining by di-8-ANEPPS. Scale bar, 50 µm (inset, 5 µm).

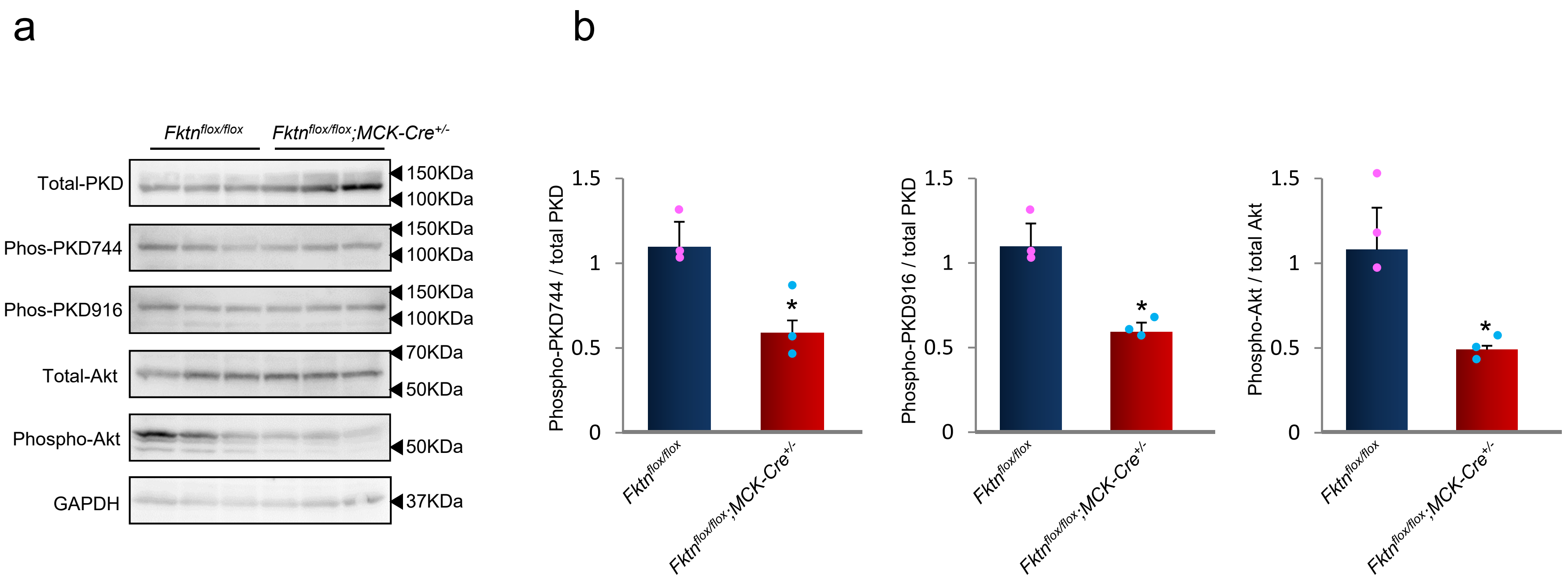

Supplementary Fig. 11: Changes in expression and the phosphorylation level of PKD and AKT in floxed (*Fktn*$^{\text{flox/flox}}$) and MCK-*Fktn*-cKO (*Fktn*$^{flox/flox}$*;MCK-Cre*$^{+/-}$) hearts at 48 weeks after birth.

(a) Representative immunoblot showing expression of total-PKD, Phospho-PKD (S744), Phospho-PKD (S916), total-AKT and phospho-AKT relative to GAPDH in 48 weeks-old of floxed or cKO hearts (N=3 per group). Although the phosphorylation level of PKD is not change in both hearts, the expression of total PKD is increased in MCK-*Fktn*-cKO hearts. The phosphorylation level is decline in in MCK-*Fktn*-cKO hearts. 10 μg protein extracts were loaded in each lane. (**b**) Expression levels of each protein. Asterisk, $P < 0.05$ versus hearts of 48-week-old floxed mice based on t-tests.

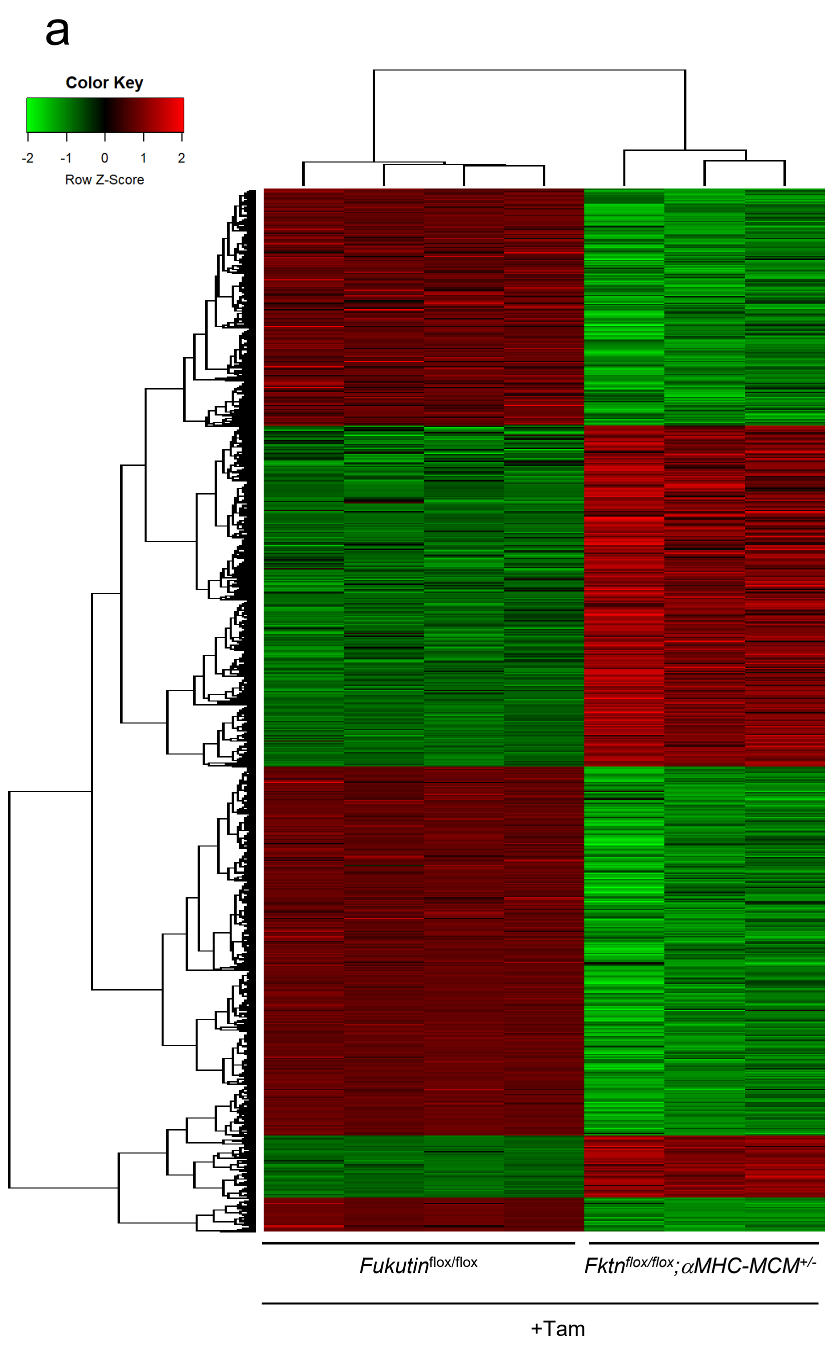

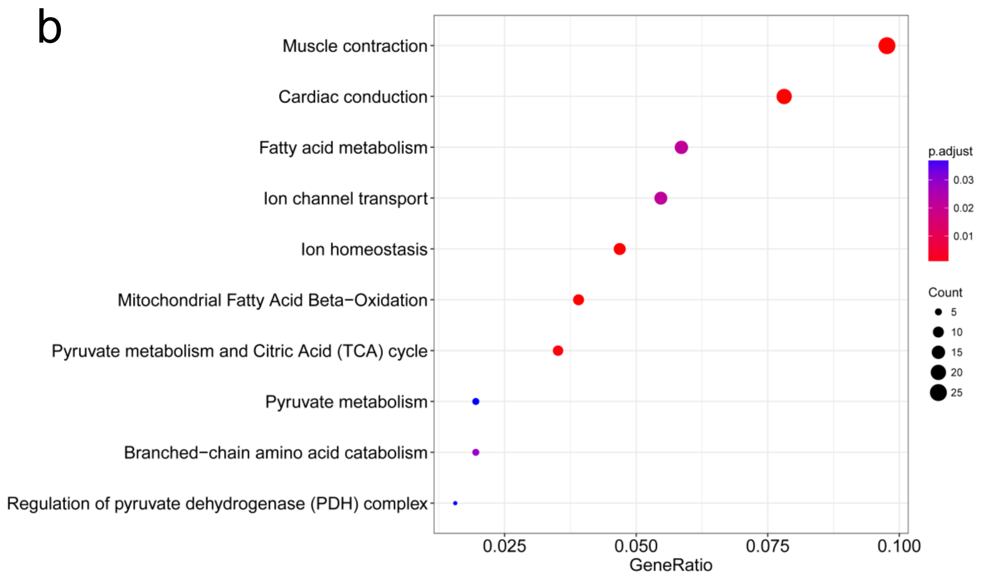

Supplementary Fig. 12: Transcriptional profile in tamoxifen-treated floxed (*Fktn*$^{flox/flox}$) and αMHC-MCM-*Fktn*-cKO (*Fktn*$^{flox/flox}$*;αMHC-MCM*$^{+/-}$) hearts (**a**) Heat map displaying hierarchical clustering and expression levels of differentially expressed in tamoxifen treated floxed and αMHC-MCM-*Fktn*-cKO hearts. 7408 genes were differentially expressed in cKO hearts based on an adjusted value of $P < 0.05$ and a > 2-fold change relative to floxed hearts. (**b**) Gene Set Enrichment Analysis revealing pathway and processes negatively enriched in tamoxifen-treated αMHC-MCM-*Fktn*-cKO hearts compared Floxed hearts.

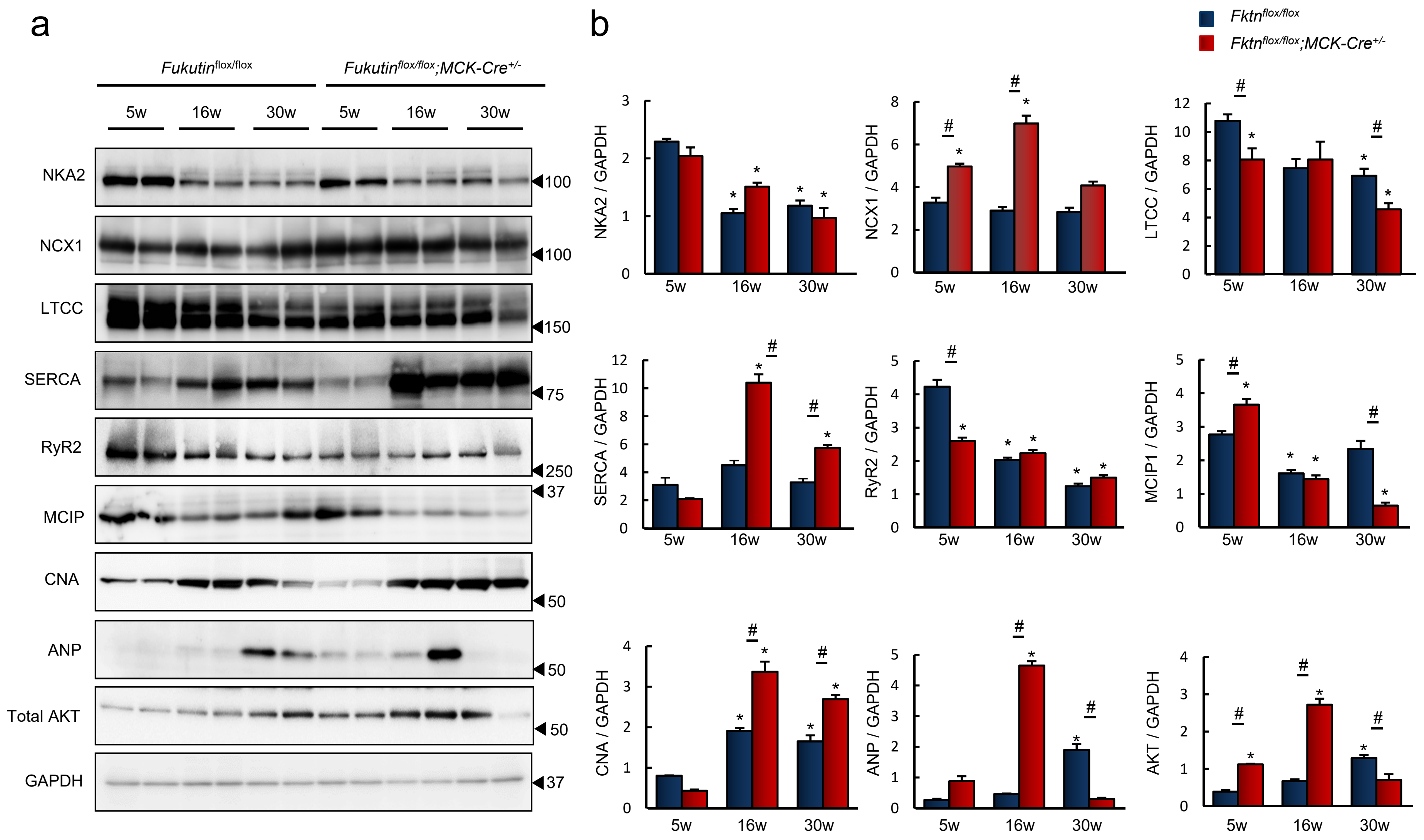

Supplementary Fig. 13: Changes in expression in floxed (*Fktn*$^{flox/flox}$) and MCK-*Fktn*-cKO (*Fktn*$^{flox/flox}$*;MCK-Cre*$^{+/-}$) hearts at different time points after birth. (**a**) Representative immunoblot showing expression of Na^+/K^+ ATPase 2 (NKA2), NCX1, LTCC, SERCA, RyR2, MCIP-1, calcineurin (CNA), ANP, and AKT relative to GAPDH in various ages of floxed or cKO hearts. In floxed control mice, NCX1, LTCC and SERCA2 expression show no great differences during postnatal development (5–30 weeks). However, *Fktn*-deficient mice showed enhanced expression of NCX1 at 5 and 16 weeks old, decreased LTCC expression at 5 and 30 weeks old, and increased expression of SERCA2 at 16 and 30 weeks old. The upregulation of CAN, downregulation of MCIP, and excessive expression of ANP suggest that MCK-*Fktn*-cKO myocytes of 16-week-old mice already have advanced hypertrophic remodeling mediated by Ca^{2+}-calcineurin signaling; 10 µg protein extracts were loaded in each lane. (**b**) Expression levels of each protein during development. Asterisk, $P < 0.05$ versus hearts of 5-week-old floxed mice; hash mark, $P < 0.05$ versus indicated groups based on Tukey-Kramer tests.

Supplementary Figure 13 Ujihara et al.,

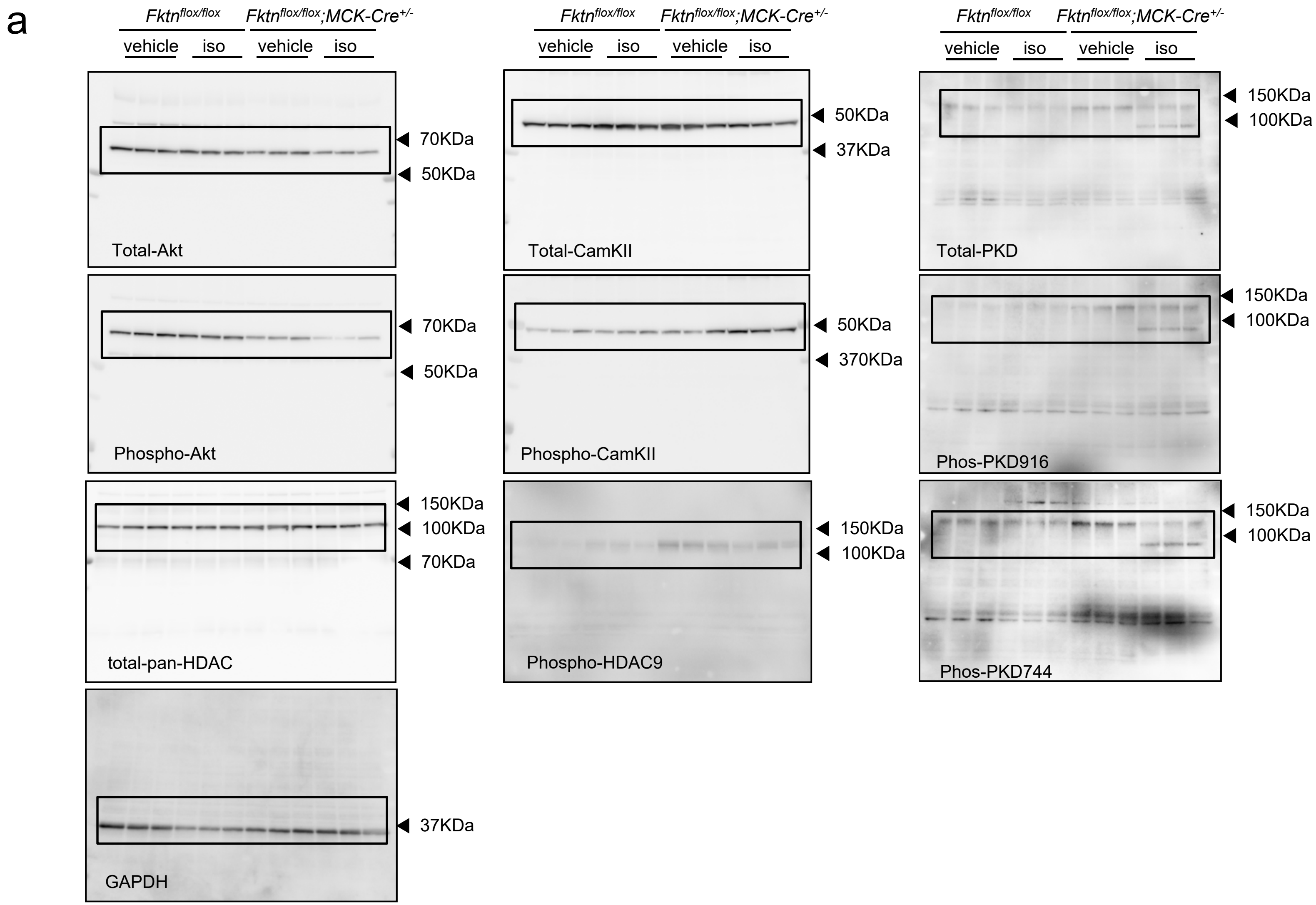

Supplementary Fig. 14: Full-length images of immunoblots for Figure 3f, 7c and Supplementary Figure 11a.
(a) Immunoblots using antibodies against Akt, phospho-Akt, CamKII, phospho-CamKII, PKD, phospho-PKD (S961), phospho-PKD (S744), HDAC, phospho-HDAC9, and GAPDH as shown in Figure 3f. For each lane, 10 μg protein extracts were loaded.

b

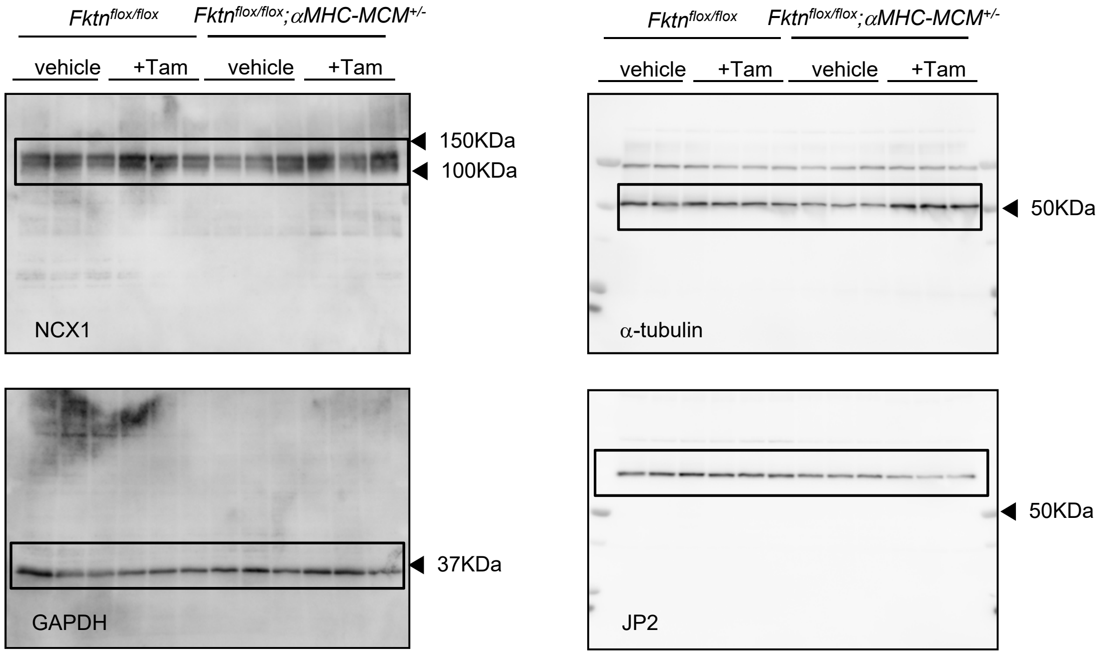

Supplementary Fig. 14 (b): Full-length images of immunoblots for Figure 6a.
(**b**) Immunoblots using antibodies against NCX1, α-tubulin, JP2, and GAPDH as shown in Figure 6a. For each lane, 10 μg protein extracts were loaded.

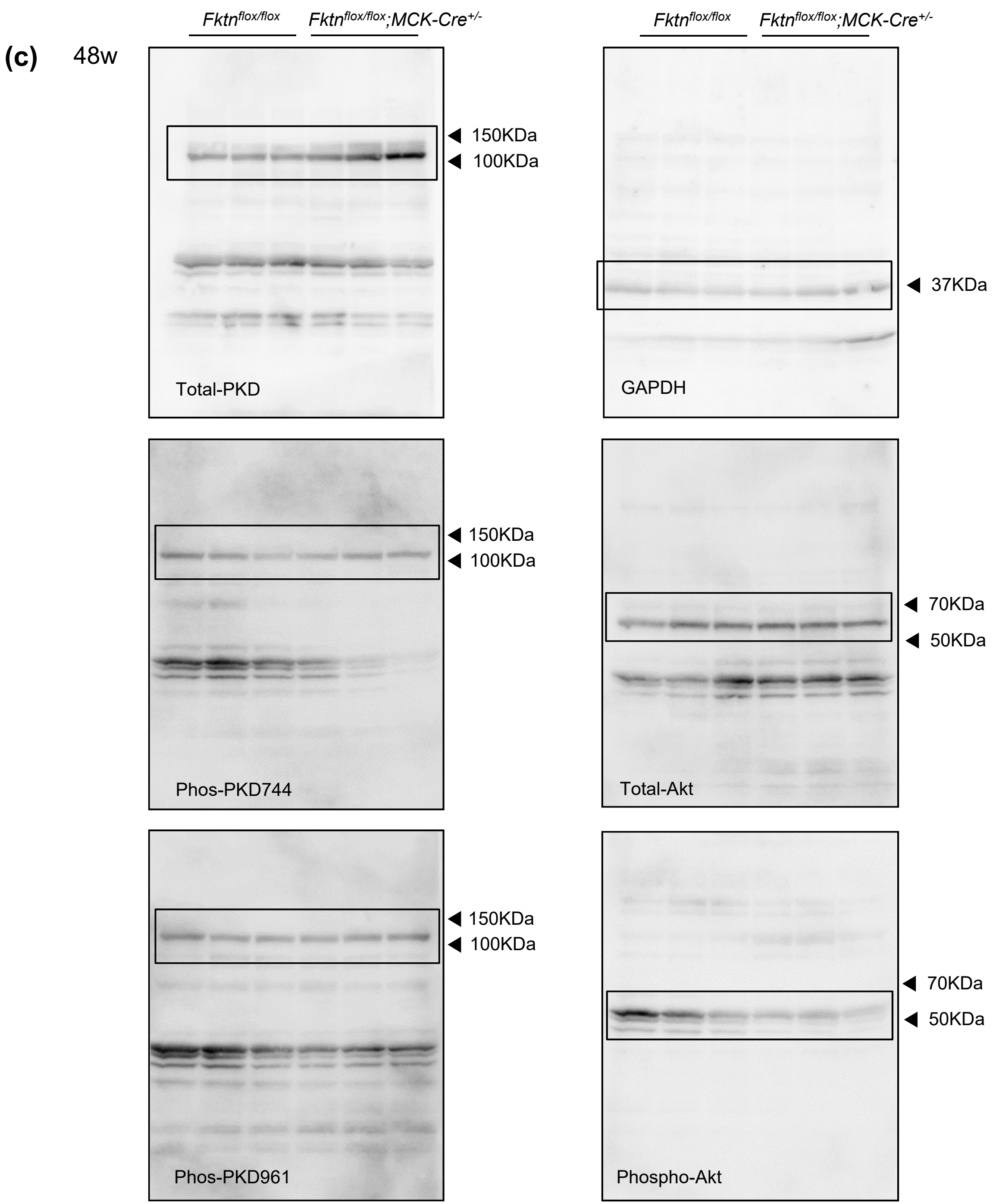

Supplementary Fig. 14 (c): Full-length images of immunoblots for Supplementary Figure 11a.
(c) Immunoblots using antibodies against PKD, phospho-PKD (S916), phospho-PKD (S744), AKT, phospho-PKD and GAPDH as shown in Supplementary Figure 11a.

Primers

Name	Sequences
Fukutin-F	GTCAAATAGCATAATTACGGGACAG
Fukutin-R	CAAGTATGGCAGTACACATTTATCG
mEx1F1	TACCCTGAGTAGCGGTCGTC
mEx2R2	GTGCTTGTAGTAGTACAGCT
mEx4R1	ACAGGTGATACTGCAGTGCG
Cre-newF	CCATCTGCCACCAGCCAG
Cre-newR	TCGCCATCTTCCAGCAGG
mGAPDH-F5	TGTVVGTCGTGGATCTGAC
mGAPDH-R5	CCTGCTTCACCACCTTCTTG

Supplementary Table 1: The name of primers and the sequences.
