# Supplementary material for: Elimination of fukutin reveals cellular and molecular pathomechanisms in muscular dystrophy-associated heart failure
Source: Nat Commun. 2019 Dec 17;10:5754. doi: 10.1038/s41467-019-13623-2 (PMC6917736; doi:10.1038/s41467-019-13623-2)
Supplement: Supplementary file 8 — Description of Additional Supplementary Files [file 41467_2019_13623_MOESM8_ESM.pdf]

Title: Supplementary Data 1.
Description: Source data underlying Fig. 1e. Cross-sectional areas of cardiomyocytes from floxed or MCK-*Fktn*-cKO hearts.

Title: Supplementary Data 2.
Description: Source data underlying Fig. 3c. Change in cross-sectional areas of cardiomyocytes from floxed or MCK-*Fktn*-cKO hearts induced by hemodynamic stress.

Title: Supplementary Data 3.
Description: Source data underlying Fig. 4c. Change in cell area of PE-treated myocytes.

Title: Supplementary Data 4.
Description: Source data underlying Fig. 4e. Estimation of SR Ca^{2+} contents.

Title: Supplementary Data 5.
Description: Source data underlying Fig. 8b. Electrical stimulation-induced (1 Hz) myocyte shortening.

Title: Supplementary Data 6.
Description: Source data underlying Fig. 8d. Normalized peak amplitude.
