# Supplementary material for: Elimination of fukutin reveals cellular and molecular pathomechanisms in muscular dystrophy-associated heart failure
Source: Nat Commun. 2019 Dec 17;10:5754. doi: 10.1038/s41467-019-13623-2 (PMC6917736; doi:10.1038/s41467-019-13623-2)
Supplement: Supplementary file 9 — Reporting Summary [file 41467_2019_13623_MOESM9_ESM.pdf]

nature research

Corresponding author(s): Yuki Katanosaka

Last updated by author(s): Sep 25, 2019

Reporting Summary

Nature Research wishes to improve the reproducibility of the work that we publish. This form provides structure for consistency and transparency in reporting. For further information on Nature Research policies, see Authors & Referees and the Editorial Policy Checklist.

Statistics

For all statistical analyses, confirm that the following items are present in the figure legend, table legend, main text, or Methods section.

n/a	Confirmed	
☐	☒	The exact sample size (*n*) for each experimental group/condition, given as a discrete number and unit of measurement
☐	☒	A statement on whether measurements were taken from distinct samples or whether the same sample was measured repeatedly
☐	☒	The statistical test(s) used AND whether they are one- or two-sided<br>*Only common tests should be described solely by name; describe more complex techniques in the Methods section.*
☒	☐	A description of all covariates tested
☐	☒	A description of any assumptions or corrections, such as tests of normality and adjustment for multiple comparisons
☐	☒	A full description of the statistical parameters including central tendency (e.g. means) or other basic estimates (e.g. regression coefficient) AND variation (e.g. standard deviation) or associated estimates of uncertainty (e.g. confidence intervals)
☒	☐	For null hypothesis testing, the test statistic (e.g. *F*, *t*, *r*) with confidence intervals, effect sizes, degrees of freedom and *P* value noted<br>*Give P values as exact values whenever suitable.*
☒	☐	For Bayesian analysis, information on the choice of priors and Markov chain Monte Carlo settings
☒	☐	For hierarchical and complex designs, identification of the appropriate level for tests and full reporting of outcomes
☒	☐	Estimates of effect sizes (e.g. Cohen's *d*, Pearson's *r*), indicating how they were calculated

Our web collection on statistics for biologists contains articles on many of the points above.

Software and code

Policy information about availability of computer code

Data collection	Cell shortoning aof isolated myocytes was assessed with either inverted microscope (IX71; Olympus). Ca2+ transients were measured using a high-performance Evolve EMCCD camera (Photometrics) and analyzed using MetaMorph software (version 7.7.1.0; Molecular Devices). SR Ca2+ contens was measured by ORCA-Flash2.8 (Hamamatsu Photonics) and analyzed using with MetaFluor software (version 7.7.5.0; Molecular Devise). Immunocytochemistry were performed using a conforcal microscope (Fluoview FV1000; Olympus) mounted on Olympus IX81 epifluorescent microscope.Immunoreactive bands were visualized using a chemiluminescence detection system (Perkin Elmer or Amersham Bioscience Corp.) and LAS3000 luminescent image analyzer (Fuji Film).
Data analysis	All data are expressed as mean±SEM and the numbers of independent experiments are indicated. Statistical comparisons were conducted between 2 groups by use of Student t-test. Multiple groups were compared with ANOVA with a post hoc Tukey–Kramer multiple comparisons test as indicated in legends. A P value <0.05 was considered significant. All statistics were done using GraphPad Prism version 6 (GraphPad Software, La Jolla, CA).

For manuscripts utilizing custom algorithms or software that are central to the research but not yet described in published literature, software must be made available to editors/reviewers. We strongly encourage code deposition in a community repository (e.g. GitHub). See the Nature Research guidelines for submitting code & software for further information.

Data

Policy information about availability of data

All manuscripts must include a data availability statement. This statement should provide the following information, where applicable:

- Accession codes, unique identifiers, or web links for publicly available datasets
- A list of figures that have associated raw data
- A description of any restrictions on data availability

The data that support the findings of this study are available from the corresponding author upon reasonable request.

Field-specific reporting

Please select the one below that is the best fit for your research. If you are not sure, read the appropriate sections before making your selection.

☒ Life sciences ☐ Behavioural & social sciences ☐ Ecological, evolutionary & environmental sciences

For a reference copy of the document with all sections, see nature.com/documents/nr-reporting-summary-flat.pdf

Life sciences study design

All studies must disclose on these points even when the disclosure is negative.

Sample size	For in vitro analysis sample size was 5 to 52. For in vivo studies sample size were 9 to 12 in each group as required for statistically significance.
Data exclusions	no data exclusion
Replication	either 3 or more times
Randomization	Floxed-control and/or littermate were used for control of knockout studies.
Blinding	All the experiment were performed blinded.

Reporting for specific materials, systems and methods

We require information from authors about some types of materials, experimental systems and methods used in many studies. Here, indicate whether each material, system or method listed is relevant to your study. If you are not sure if a list item applies to your research, read the appropriate section before selecting a response.

Materials & experimental systems

n/a	Involved in the study	
☐	☒	Antibodies
☐	☒	Eukaryotic cell lines
☒	☐	Palaeontology
☐	☒	Animals and other organisms
☒	☐	Human research participants
☒	☐	Clinical data

Methods

n/a	Involved in the study	
☒	☐	ChIP-seq
☒	☐	Flow cytometry
☒	☐	MRI-based neuroimaging

Antibodies

Antibodies used	Primary antibodies were obtained from Cell Signaling (AKT #9272, phospho-AKT #4060,PKD #2052, phospho-PKD (S916) #2051, (S744) #2054, MEF2 #5030), Santacruz (Dystrophin #sc-15376, JP2 #sc-51313, CamKII #sc-9035, phospho-CamKII #sc-12886), Leica (alpha-SG #20A6, beta-SG #5B1, gamma-SG #2185, beta-DG #B-DG-CE), Abcam (GAPDH #ab-9484, HDAC9 #7628, MCIP #ab-25124, GM130 #ab-52649), BD Biosciences (Cav3 #610421), Millipore (IIH6 #05-593), phospho-Foxo3a (Thr32) #9464, phospho-Foxo3a (Ser253) #9466, JNK #9252, phospho-JNK #9251), Phoenix (ANP #H-005-24), Alomone (LTCC #AC-003), Echelon (PI4P #Z-P004), Sigma (alpha-tubulin #T5168, phospho-HDAC9 #SAB4300269) and Thermofisher scientific (NAK #07-674, SERCA2 #MA#-901, RyR #MA3-916). The anti-NCX antibody was generated in our laboratory.
Validation	We used negative control in each experiment.

Eukaryotic cell lines

Policy information about cell lines

Cell line source(s)	All the cell lines were purchased from ATCC. Primary cells were isolated as described in material methodsAll the cell lines were purchased from ATCC. Primary cells were isolated as described in material methods
Authentication	As described by ATCC
Mycoplasma contamination	Mycoplasma kit were used
Commonly misidentified lines (See ICLAC register)	*Name any commonly misidentified cell lines used in the study and provide a rationale for their use.*

Animals and other organisms

Policy information about studies involving animals; ARRIVE guidelines recommended for reporting animal research

Laboratory animals	All the animals were back crossed to C57BL/J. Mice were purchased from Jackson laboratory. Floxed-Fktn mice were genereted in Kobe University. MCK-cre (#006475) and MerCreMer-MHC-cre (#005650) mice were purchased from Jackson Laboratory and crossed with conditional Fktn-allele (Floxed-Fktn) mice.
Wild animals	All the mice were of C57BL/J background.
Field-collected samples	*For laboratory work with field-collected samples, describe all relevant parameters such as housing, maintenance, temperature, photoperiod and end-of-experiment protocol OR state that the study did not involve samples collected from the field.*
Ethics oversight	All the mouse experiment have been done according to the all relevant ethical regulations. Mice were housed in a facility accredited by the Japanese Act on the Welfare and Management of Animals (No. 105). All animal studies were approved by the Institutional Animal Care and Use Committee of the University of Okayama university.

Note that full information on the approval of the study protocol must also be provided in the manuscript.
